# Epithelial periostin expression is correlated with poor survival in patients with invasive breast carcinoma

**DOI:** 10.1371/journal.pone.0187635

**Published:** 2017-11-21

**Authors:** Ga-Eon Kim, Ji Shin Lee, Min Ho Park, Jung Han Yoon

**Affiliations:** 1 Department of Pathology, Chonnam National University Medical School, Gwangju, Republic of Korea; 2 Department of Surgery, Chonnam National University Medical School, Gwangju, Republic of Korea; University of South Alabama Mitchell Cancer Institute, UNITED STATES

## Abstract

Invasion and metastasis are direct causes of mortality in patients with breast cancer and require reciprocal interactions between cancer cells and the extracellular matrix (ECM). Periostin, a fasciclin-containing adhesive ECM glycoprotein, is frequently overexpressed in various types of human cancer, and its overexpression in cancer-associated stroma and/or cancer cells is usually associated with poor clinical outcomes. However, the expression of periostin in the successive steps of breast tumorigenesis and its association with outcome variables have not been well established in breast carcinoma. The present study aimed to assess the role of periostin alteration in breast tumorigenesis and evaluate the putative prognostic value of periostin as a function of its compartmentalization. Immunohistochemical staining with anti-periostin antibody was performed in a total of 300 patients (26 patients with normal breast tissues, 76 patients with ductal carcinoma in situ [DCIS], and 198 patients with invasive breast carcinoma [IBC]) using tissue microarray. Periostin immunoreactivity was assessed in both epithelial tissue and the surrounding stromal compartment. The mRNA and protein expression of periostin were analyzed in 10 paired normal/invasive cancer frozen specimens by quantitative real time-polymerase chain reaction and western blot analysis, respectively. In cancer tissues, periostin mRNA and protein expression were increased compared with adjacent normal tissues. Both epithelial and stromal periostin staining scores significantly increased in a stepwise manner with disease progression from normal breast tissue to DCIS and IBC (P = 0.000 and 0.000, respectively). High epithelial and stromal periostin expression was observed in 109/189 (57.7%) and 158/189 (83.6%) cases of IBC, respectively. High epithelial periostin expression was more frequently observed in the distant metastatic relapse-positive group than in the distant metastatic relapse-negative group (41/51 [80.4%] vs. 68/138 [49.3%] cases [P = 0.000]). Furthermore, high epithelial periostin expression was associated with reduced disease-free survival and overall survival in univariate and multivariate analysis. Periostin may play an important role in the progression of breast tumor, and epithelial periostin expression may serve as a new parameter for prediction of prognosis in patients with IBC. Further studies examining periostin expression and its potential as a target of IBC therapy are warranted.

## Introduction

Breast cancer is the second most common malignancy among Korean women, after thyroid cancer and is one of the leading causes of cancer death among women [1]. Breast carcinoma involves abnormal epithelial cell growth, potentially resulting in ductal carcinoma in situ (DCIS), which can develop into invasive breast carcinoma (IBC). This process may result in metastatic disease [2]. Despite recent developments in breast cancer treatment, invasion and metastasis are the critical causes of death in patients with breast cancer [3].

Tumor invasion and metastasis is a multidirectional, controlled, and complicated process. To metastasize, primary tumor cells invade local tissues, enter the blood or lymphatic circulatory system and survive, travelling to a distant secondary organ; following extravasation, the cells need to survive in a new environment [4]. In this multistep process, tumor microenvironment, composed of cellular and extracellular components, plays an important role. Reciprocal interactions between epithelial cells and mesenchymal cells, and between cancer cells and the extracellular matrix (ECM), could stimulate invasion and metastasis of cancer cells [5–7]. Alterations in the ECM components within the tumor microenvironment have a significant impact on the process of cancer progression and metastasis [8].

Periostin is a fasciclin-containing adhesive ECM glycoprotein commonly found in collagen rich connective tissues and upregulated in the embryonic periosteum, placenta, cardiac valves, and periodontal ligaments, as well as in many adult tissues, and its deposition is augmented in tissues with stress conditions and wound repair [9–11]. Reports have indicated that periostin is involved in cancer cell survival, epithelial–mesenchymal transition (EMT), ECM degradation, invasion, and distant metastasis [12–14].

Recently, it has been reported that periostin plays an important role in tumorigenesis of a wide variety of cancers, such as non-small-cell lung cancer [15], colorectal cancer [16], head and neck cancer [17], ovarian cancer [18], pancreatic cancer [19], penile cancer [20], prostate cancer [21], hepatocellular carcinoma [22], and cholangiocarcinoma [23]. Periostin expression in these cancers is well correlated with poor prognostic tumor features and poor outcome [15–23].

A limited number of studies have investigated periostin expression in breast cancer [24–29]. Periostin expression is increased in breast cancer tissues compared with normal tissues [24–29]. However, it is not clear whether periostin is located in cancer cells or cancer-associated stroma or in both compartments. Although overexpression of periostin in cancer-associated stroma and/or in cancer cells has been associated with poor clinical outcome in various types of cancer [15–23], its influence on the clinical outcome of breast cancer has received little attention. To the best of our knowledge, only one study has directly compared, although on a retrospective basis, periostin overexpression in breast cancer with clinical outcome, namely with disease-specific survival [29]. Furthermore, there have been few studies examining the role of periostin alteration in DCIS, a precursor, albeit non-obligatory, to IBC [28].

To assess the role of periostin alteration in breast tumorigenesis and to evaluate the putative prognostic value of periostin as a function of its compartmentalization, immunohistochemical staining with anti-periostin antibody was performed using tissue microarray in a total of 300 patients. Of these 26 patients had normal breast tissue, 76 patients had DCIS, and 198 patients had IBC. Periostin immunoreactivity was assessed in both epithelial and surrounding stromal compartment.

## Materials and methods

### Periostin mRNA and protein expression in breast carcinoma (BC) tissues and corresponding normal breast tissues

#### Collection of samples

Frozen samples and their corresponding formalin-fixed paraffin-embedded (FFPE) samples composed of breast carcinoma (BC) and matched normal breast tissues were provided by the Biobank of Chonnam National University Hwasun Hospital, a member of the Korea Biobank Network. We included 8 patients and informed consent was obtained from all of these participants. The resected specimen in a mirror imaged fashion alternatively submitted for biobanking and for histological assessment to assess overall appropriateness of frozen banking tissues. Approximately 1.0 x 1.0 x 0.5 cm of each BC and normal tissues were removed from the resected samples. Specimens for biobanking were stored in smaller fragments within 30 min after resection in a liquid nitrogen freezer at -196°C. Frozen samples were used for by quantitative real time-polymerase chain reaction (qRT-PCR) and Western blot analysis.

#### Quantitative real-time polymerase chain reaction (qRT-PCR)

The expression of periostin mRNA was analyzed by qRT-PCR, as previously described [25]. Total RNA was extracted from frozen breast tissues using a RNeasy Mini Kit (Qiagen, Valencia, CA) and used to prepare cDNA synthesis by GoScript^TM^ Reverse Transcription System (Promega, Madison, WA). The real-time PCR reaction was performed with TaqMan^®^ Gene Expression Maser Mix (Thermo Fisher Scientific, Rockford, IL) according to the manufacturer’s instructions and with the following cycling conditions: initial denaturation for 30 s at 95°C, followed by 40 cycles of 95°C for 15 s and 60°C for 60 s, in a 7500 Fast Real-Time PCR System (Applied Biosystems, Foster City, CA). The data were analyzed using the 7500 system SDS software program (v2.0.5; Applied Biosystems). The following probes of TaqMan^®^ Gene Expression Assays (Thermo Fisher Scientific) were used: Hs001566734_m1 (periostin) and Hs02758991_g1 (glyceraldehyde 3-phosphate dehydrogenase, GAPDH). All experiments were performed in triplicate. The 2^-ΔΔCt^ method was used for data analysis [26]. The value of 2^-ΔΔCt^ indicated the fold change in gene expression normalized to GAPDH.

#### Western blot analysis

The level of periostin protein was determined by Western blot analysis, as described previously [29]. For immunolabeling, mouse polyclonal antibodies to periostin (1:1,000 dilution, US Biological Life Sciences, Salem, CA) and β-actin antibody (1: 1,000 dilution; Abcam; rabbit polyclonal) as an internal loading control were used. For quantification, the bands for periostin and β-actin protein were measured using Multi gauge V3.0 analysis software (Fujifilm Life-science, Tokyo, Japan).

### Periostin expression in normal breast, DCIS, and IBC tissues

#### Tissues and patients

Archival paraffin-embedded tissue from normal breast tissue (n = 26), DCIS (n = 76), and IBC (n = 198) were retrieved from the files of the Department of Pathology, Chonnam National University Hospital and Chonnam National University Hwasun Hospital, Korea. Normal breast tissue samples were taken from women with no surgically proven pathological breast lesions. Some of the specimens were provided by the Chonnam National University Hwasun Hospital National Biobank of Korea (07SA2015001-001), a member of the National Biobank of Korea.

We selected 198 IBC cases with a minimum follow-up of 10 years. Complete outcome data, including survival status, survival time, cause of death, disease-free interval, time to loco-regional recurrence, and/or distant metastasis, were available for all patients. Patient’ clinical history and cancer characteristics, including patient age at the initial diagnosis, histologic type, tumor size, nodal status, Nottingham combined histologic grade, stage, hormone receptor status [estrogen receptor-α (ER-α) and progesterone receptor (PR)], and epidermal growth factor receptor HER2/neu expression, were available for all patients. Luminal, HER2, and triple negative surrogates of molecular subtypes were defined according to expression of ER-α, PR, and HER2.

No IBC patients had received previous chemotherapy or radiotherapy. Adjuvant chemotherapy and radiotherapy were performed in 165 (83.3%) and 106 (51.5%) patients, respectively. One hundred and thirty-eight patients (69.7%) underwent hormone therapy. Sixty-one patients experienced local recurrence or metastasis (12 with local recurrence and 51 with distant metastases), and 138 remained disease free. There were 50 deaths due to breast carcinoma.

The Institutional Review Board (IRB) at the Chonnam National University Hwasun Hospital (Jeollanam-do, Korea) approved the study protocol (CNUHH-2014-156) and provided all the necessary ethical approvals.

#### Tissue microarray construction

Tissue microarray (TMA) construction was performed on 1 representative paraffin block from each case. In brief, tissue 1.5 mm diameter cores were punched from representative tumor regions of each donor block of IBC and arrayed into a new recipient paraffin block using a tissue microarrayer (Beecher Instruments, Sun Prairie, WI). Each sample was arrayed in triplicate. Tissue cores of 2.0mm in diameter were punched from the representative regions of each donor block of normal breast tissues and DCIS. Each sample was arrayed in duplicate.

#### Immunohistochemistry

Automated immunohistochemical staining was performed using the Bond-max system (Leica Microsystems, Bannockburn, IL). Briefly, following deparaffinization, rehydration, and heat-induced epitope retrieval (antigen unmasking) with Bond Epitope Retrieval Solution 1 (Leica Microsystems) for 20 minutes at 100°C, the tissue microarray tissue sections were incubated with the primary antibody directed against periostin (Abcam; rabbit polyclonal; dilution 1:150) for 15 minutes at room temperature. Normal human serum served as a negative control.

#### Evaluation of immunohistochemical staining

Tissue microarrays were digitized (Aperio Technologies, Vista, CA) and immunoreactivity was evaluated. Evaluation of all immunohistochemical staining was performed as a blind assessment and independently by two investigators. Any discordant findings between the two observers were settled using a multi-headed microscope. Periostin immunoreactivity in the epithelial compartment (inner luminal cells of normal breast or tumor cells of DCIS and IDC) and in the surrounding stromal compartment was evaluated using the intensity and extent of staining. Briefly, the intensity of staining was scored on a scale of 0 to 3 (0 = negative staining, 1 = weakly positive staining, 2 = moderately positive staining, and 3 = strongly positive staining). The intensity of staining was determined according to the maximum intensity of staining observed in the case. The extent of positive staining was estimated and scored on a scale of 0 to 4 (0 = negative, 1 = <10% positive cells, 2 = 10 to <30% positive cells, 3 = 30 to <50% positive cells, and 4 = >50% positive cells). The sum of the intensity score and the extent of staining score were used as the final staining scores (SS).

In IBC, cases with epithelial SS of <4 were categorized in the low expression group, and cases with SS of 4 to 7 were categorized in the high expression group. Cases with stromal SS of <6 were categorized in the low expression group, and those with SS of 6 to 7 were categorized in the high expression group. These cut-off values were the values that better discriminated the patient-cohort according to the main clinical outcome endpoints of the study.

#### Statistical analysis

One-way ANOVA and *t*-test were used to assess the difference in the periostin SS between normal breast, DCIS, and IBC. Spearman correlations (r) were calculated to evaluate the correlation in periostin SS between epithelial and stromal compartment in each group. The association between clinicopathological features of the patients with IBC and expression of periostin was analyzed using the χ^2^ test. Survival curves were constructed using the Kaplan-Meier method. The distribution of survival was compared using the log-rank test. Multivariate analysis was performed using Cox’s proportional hazard model. For all statistical analyses, the SPSS system for personal computer (version 13.5 for windows; SPSS INC., Chicago, IL) was used and P < 0.05 was considered significant.

## Results

### Periostin mRNA and protein expression in BC tissues and corresponding normal breast tissues

**Fig 1:**
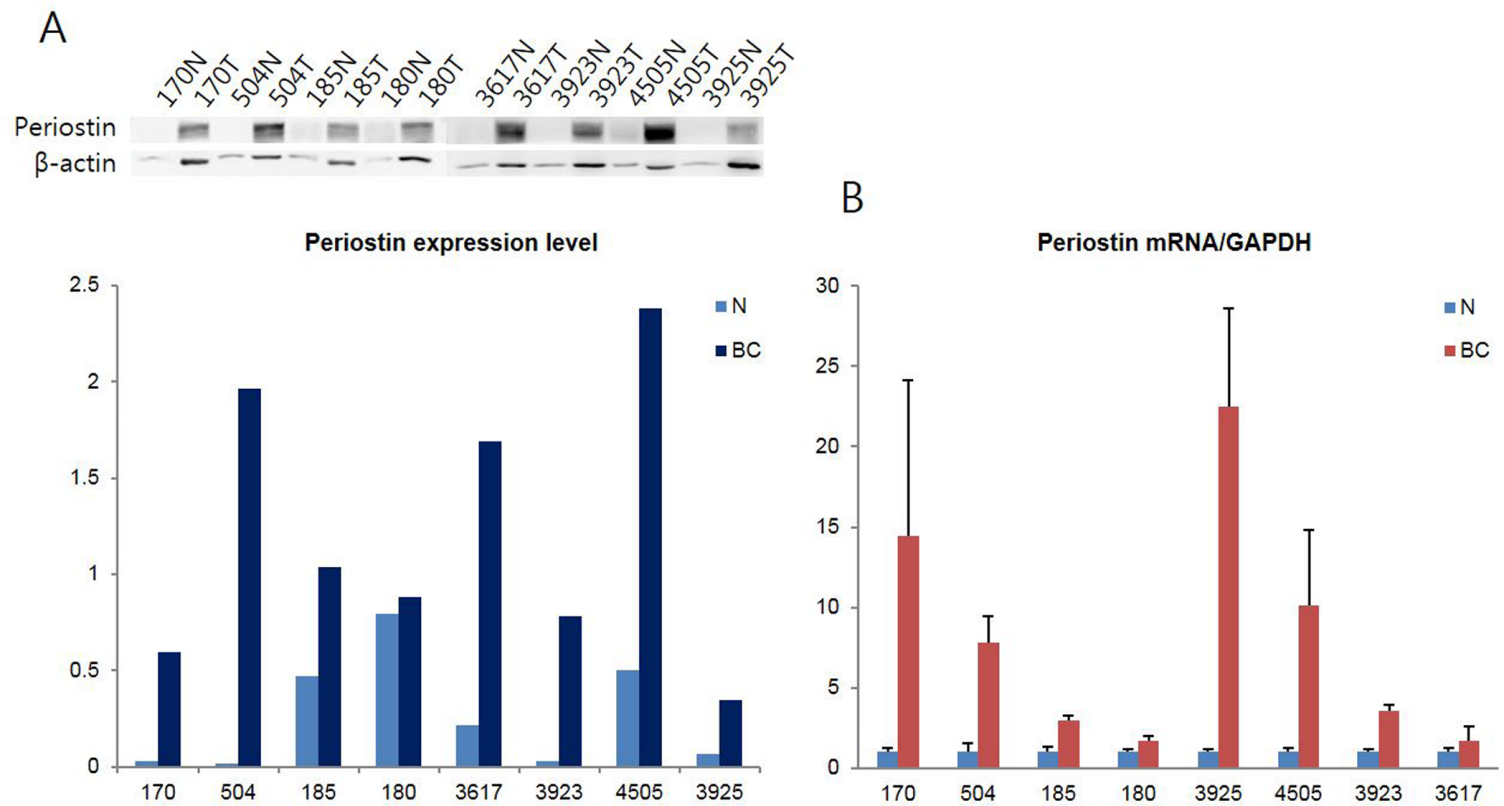
Increased periostin expression in breast carcinoma. Western blot and qRT-PCR analysis of Periostin were performed in eight paired breast carcinoma and adjacent normal specimens. **(A)** Periostin protein was increased in breast carcinoma (BC) tissues compared to that in the corresponding adjacent normal (N) breast tissues. **(B)** qRT-PCR also demonstrated up-regulated periostin mRNA expression in the BC tissues compared to N breast tissues.

We examined periostin mRNA expression by qRT-PCR analysis in eight frozen BC tissues versus surrounding normal breast tissues obtained from the same patients. Periostin mRNA was detected in all studied tissues samples. As shown in Fig 1, the expression level of periostin mRNA in breast carcinoma tissues was elevated compared with that in corresponding normal tissues and the difference was significant 8.12 ± 7.37 vs. 1.00 ± 0.00, P < 0.05). To find out the correlation of periostin mRNA expression with protein levels, periostin protein levels was measured by western blotting in the same samples used for qRT-PCR analysis. The density of periostin expression measured by quantitative analysis was significantly increased in BC tissues compared with the corresponding normal breast tissues (1.21 ± 0.72 vs. 0.27 ± 0.29, P < 0.01). Periostin mRNA and protein ratio between BC tissues and corresponding normal tissues in all 8 patients with BC was higher than 1. There was a positive correlation between the periostin mRNA BC/normal ratio and periostin protein BC/normal ratio (r = 0.690, P < 0.05).

### Periostin expression in normal and neoplastic breast tissues

**Fig 2:**
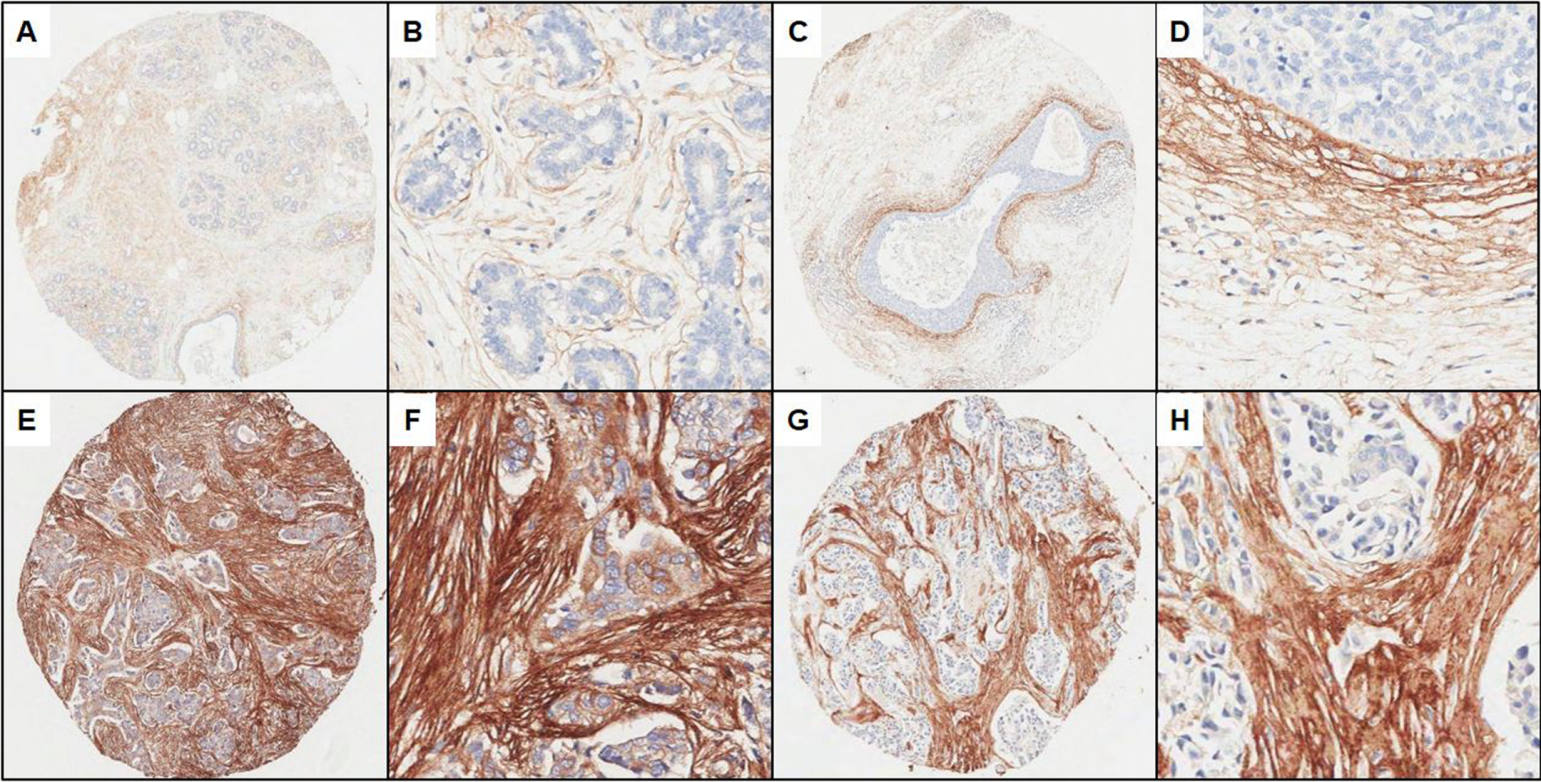
**Periostin expression in normal breast tissue (A,B), ductal carcinoma in situ (C,D), and invasive breast carcinoma (E-H) TMA cores.** A, C, E, G: low magnification; B, D, F, H: high magnification.

Immunostaining data of periostin were available in 26/26 (100%) cases of normal breast tissue, 74/76 (97.4%) cases of DCIS, and 189/198 (95.5%) cases of IBC, after the exclusion of non-informative and missing cores (Fig 2).

In all normal breast tissue, periostin expression was restricted to the stromal connective tissue, while the epithelial component was devoid of periostin (Fig 2A and 2B). The intralobular or interlobular stromal compartment displayed a fibrillary pattern of periostin staining with considerable variation of staining intensity (Figure A in S1 File). In the majority of cases, periostin was faintly expressed and confined to a narrow faint band adjacent to the ducts. In some cases, normal ducts were surrounded by well-defined bundles of periostin-positive stromal tissues. In DCIS and IBC, periostin was occasionally detected in the cytoplasm of the malignant epithelial cells (Figures B and C in S1 File). In DCIS lesions, there were variable and discrete regions of periostin-positive stroma immediately adjacent to the malignant epithelium. Periostin-positive stroma began to expand and formed a broad, well-defined band at the epithelial-stromal interface (Fig 2C and 2D). However, stromal periostin staining in IBC was more diffuse and intense, rather than juxtaposed to malignant epithelium as seen in DCIS lesions (Fig 2E–2H).

The SS of periostin in the epithelial and stromal compartment in normal breast tissue, DCIS, and IBC are summarized in Table 1. Epithelial and stromal SS increased significantly with disease progression in a stepwise fashion from normal breast tissues, to DCIS and then to IBC (P = 0.000 and P = 0.000, respectively). Epithelial SS were significantly higher in IBC than in normal tissue or DCIS (P = 0.000 and P = 0.000, respectively). Epithelial SS were also significantly higher in DCIS than in normal breast tissue (P = 0.000). Stromal periostin SS were significantly higher in IBC than in either normal tissue or DCIS (P = 0.000 and P = 0.000, respectively), while the difference in SS between normal tissue and DCIS was not significant.

**Table 1:** Staining scores of periostin expression in normal and neoplastic breast tissues.

Histologic stage	No. of cases	Epithelial compartment (Mean ± SD)	*P* value	Stromal compartment (Mean ± SD)	*P* value
Normal	26	0	< .001*	4.64 ± 0.48	< .001*
Ductal carcinoma in situ	74	2.22 ± 1.65		4.89 ± 0.62	
Invasive breast carcinoma	189	4.06± 1.83		6.44 ± 0.88	

N, numbers

*Linear-by-linear association

Periostin SS in the stromal compartment were significantly higher than those observed in the epithelial compartment in each group (normal breast, P = 0.004; DCIS, P = 0.000; and IBC, P = 0.000). Notably, stromal periostin SS significantly correlated with epithelial periostin SS in DCIS and IBC (DCIS, r = 0.297, P = 0.010; IBC, r = 0.207, P = 0.004).

### Association between periostin expression and clinicopathological features of IBC

On the basis of the cut-off point, we observed high epithelial and stromal periostin expression in 109/189 (57.7%) and 158/189 (83.6%) of cases of IBC. The association between periostin expression and clinicopathological characteristics is summarized in Table 2. Clinicopathological factors were compared between the periostin high expression group and the low expression group, and no significant difference was found except for metastatic relapse. High epithelial periostin expression was more frequent in the distant metastatic relapse-positive group than in the distant metastatic relapse-negative group [41/51 (80.4%) vs. 68/138 (49.3%) cases, respectively (P = 0.000)]

**Table 2:** Association between periostin expression and clinicopathological parameters in invasive breast carcinomas.

Characteristics	High epithelial periostin expression N/total N (%)	*P* value*	High stromal periostin expression N/total N (%)	*P* value*
**Age (years)**		1.000		0.053
≤ 46	61/106(57.5)		83/106(78.3)	
> 46	48/83 (57.8)		75/83 (90.4)	
**Histopathologic type**		0.556		0.459
Invasive ductal carcinoma, NOS	96/162 (59.3)		137/162 (84.6)	
Invasive lobular carcinoma	12/25 (48.0)		19/25 (76.0)	
Mucinous carcinoma	1/2 (50.0)		2/2 (100)	
**Tumor size (cm)**		0.655		0.226
≤ 2	22/32 (68.8)		24/32 (75.0)	
2–5	65/128 (50.8)		109/128 (85.2)	
> 5	22/29 (75.9)		25/29 (86.2)	
**Number of nodal metastasis**		0.229		0.257
0	57/101 (56.4)		82/101 (81.2)	
1–3	27/48 (56.3)		40/48 (83.3)	
4–9	11/24 (45.8)		22/24 (91.7)	
≥ 10	14/16 (87.5)		14/16 (87.5)	
**Histologic grade**		0.579		0.462
1	13/23 (56.5)		22/23 (95.7)	
2	54/97 (55.7)		78/97 (80.4)	
3	42/69 (60.9)		58/69 (84.1)	
**Stage**				
I	22/32 (68.8)	0.787	24/32 (75.0)	0.080
II	54/108 (50.0)		90/108 (83.3)	
III	33/49 (67.3)		44/49 (89.8)	
**Estrogen receptor- α**		0.140		1.000
Negative	53/83 (63.9)		69/83 (83.1)	
Positive	56/106 (52.8)		89/106 (84.0)	
**Progesterone receptor**		1.000		0.166
Negative	49/85 (57.6)		75/85 (88.2)	
Positive	60/104 (57.7)		83/104 (79.8)	
**HER-2**		0.061		1.000
Negative	83/153 (54.2)		128/153 (83.7)	
Positive	26/36 (72.2)		30/36 (83.3)	
**Chemotherapy/radiotherapy**		0.022		0.401
No	10/27 (37.0)		21/27 (77.8)	
Yes	99/162 (61.1)		137/162 (84.6)	
**Molecular subtypes**		0.211		0.243
Luminal	73129 (56.6)		104/129 (80.6)	
HER2 Triple negative	15/20 (75.0) 21/40 (52.5)		18/20 (90.0) 36/40 (90.0)	
**Hormonal therapy**		0.155		0.394
No	38/58 (65.0)		51/58 (87.9)	
Yes	71/131 (54.2)		107/131 (81.7)	
**Metastatic relapse**		< .001		1.000
No	68/138 (49.3)		115/138 (83.3)	
Yes	41/51 (80.4)		43/51(84.3)	

N, number; NOS, Not otherwise specified; ns, not significant

*Analyzed by χ^2^ test

### Summary of survival analysis

**Fig 3:**
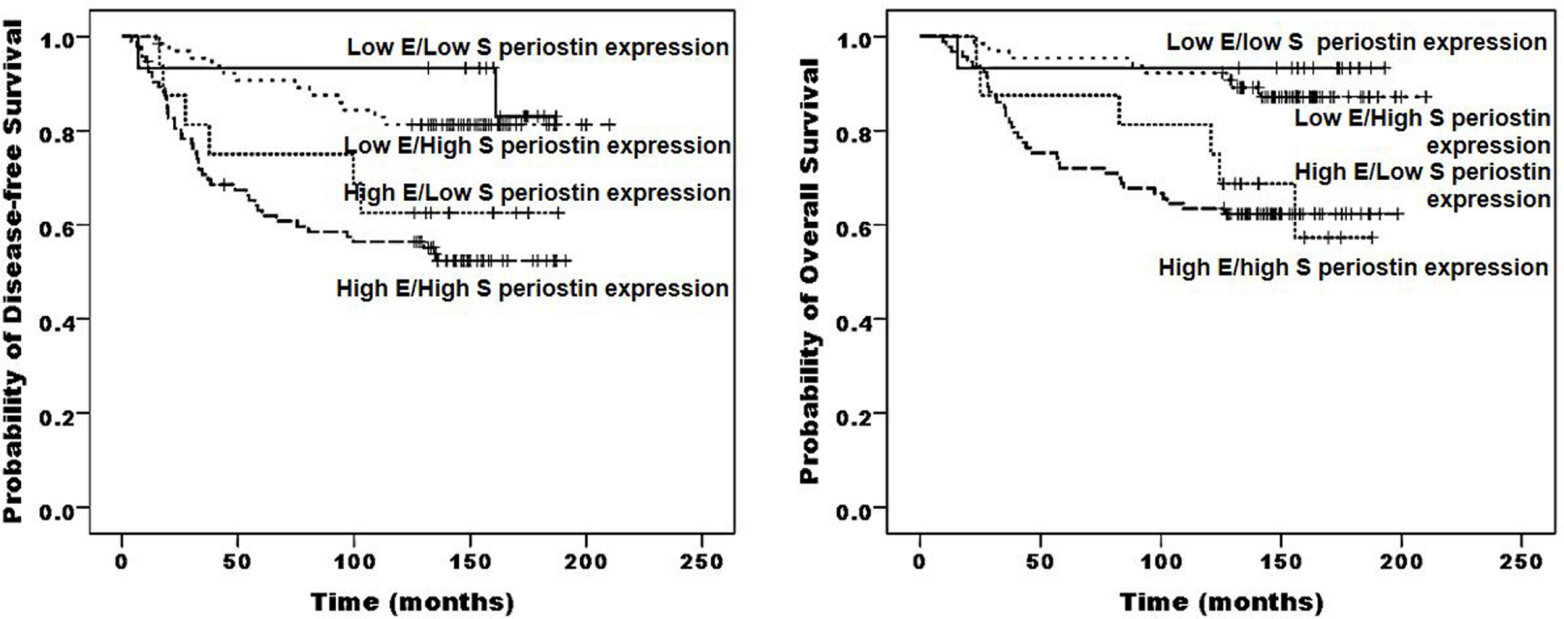
Survival of patients grouped according to periostin expression. The categorization of periostin expression as high or low for both the epithelial and the stromal compartment cannot predict significantly disease-free and overall survival.

On univariate analysis, tumor size, lymph node status, stage, and chemotherapy/radiotherapy status were significant prognostic factors for a disease-free state as well as for overall survival (Table 3). The disease-free and overall survival curves stratified by periostin status are shown in Fig 3. Patients with high epithelial periostin expression had a significantly poorer prognosis for disease-free and overall survival than those with low periostin expression (P = 0.000 and P = 0.000, respectively). We categorized periostin expression as high or low for both the epithelial and the stromal compartment, according to the following combinations: epithelial low/stromal low, epithelial high/stromal low, epithelial low/stromal high, and epithelial high/stromal high. This categorization of periostin expression did not improve statistical performance for the estimation of survival (data not shown).

**Table 3:** Univariate analysis of prognostic factors in patients with invasive breast carcinoma.

	Survival	
	Disease-free	Overall
**Age**	0.921	0.490
**Histologic type**	0.084	0.439
**Tumor size**	0.001	< .001
**Lymph node status**	< .001	< .001
**Histologic grade**	0.533	0.110
**Stage**	< .001	< .001
**Hormonal therapy**	0.386	0.310
**Chemotherapy/radiotherapy**	0.027	0.017
**Estrogen receptor- α status**	0.446	0.411
**Progesterone receptor status**	0.565	0.658
**HER-2 status**	0.911	0.667
**Periostin epithelial expression (low vs. high)**	< .001	< .001
**Periostin stromal expression (low vs. high)**	0.291	0.547

All statistically significant variables from univariate analysis, as well as age, were entered into the multivariate Cox regression analysis (Table 4). In multivariate analysis, lymph node status and periostin epithelial expression were shown as independent prognostic factors for disease-free and overall survival.

**Table 4:** Multivariate analysis with Cox’s proportional hazard model for prognostic factors in patients with invasive breast carcinoma.

	Disease-free survival			Overall survival		
	HR	95% CI	*P* value	HR	95% CI	*P* value
**Age (≤ 46 vs. >46 years)**	0.20	0.53–1.48	0.654	0.00	0.56–1.18	0.987
**Tumor size (≤ 5 vs. >5 cm)**	0.93	0.36–1.42	0.336	0.21	0.40–1.76	0.648
**Lymph node status (negative vs. positive)**	9.79	0.17–0.66	0.002	7.49	0.14–0.72	0.006
**Stage (I/II vs. III)**	0.59	0.38–1.52	0.440	2.48	0.25–1.17	0.116
**Chemotherapy/radiotherapy (No vs. Yes)**	0.33	0.25–2.13	0.566	0.53	0.13–2.54	0.467
**Periostin epithelial expression (low vs. high)**	13.71	0.17–0.58	< .001	13.00	1.87–8.16	< .001

## Discussion

Periostin expression by cancer cells and/or cancer-associated stroma has been found to correlate with tumor progression and clinical outcome in various types of cancer. In this study, we demonstrated increased epithelial and stromal periostin expression with progression of histology along the spectrum of normal-DCIS-IBC. Epithelial periostin expression was one of the independent prognostic factors for disease-free and overall survival in patients with IBC.

ECM is defined as a complex mixture of proteins that provides structural and biochemical support to the connective tissues. Reciprocal interactions between cancer cells and ECM sustain tumor growth and angiogenesis, degrade the ECM, and enhance tumor cell invasion and metastasis [5–8]. Periostin, a unique ECM protein, is physiologically expressed in a wide variety of normal adult tissues and fetal tissues and plays an important role, regulating cell function and cell-matrix interactions [9–11]. Recent studies revealed that periostin is involved in tumor development and progression [12–14] and its overexpression in cancer-associated stroma and/or in cancer cells has been associated with poor clinical outcome in various types of human cancer [15–23].

Breast tumor progression involves a complex series of processes, including induction of angiogenesis, invasion of neighboring tissues, and metastasis to the distant organs. Current reports have demonstrated that periostin plays a critical role in the acquisition of most of these hallmarks of breast tumor progression. Overexpression of periostin in human breast cancer cells leads to a significant enhancement of angiogenesis [25]. Human MDA-MB-231 breast cancer cells engineered to overexpress periostin showed a phenotype of accelerated angiogenesis as xenografts in immunocompromised animals. The underlying mechanism of periostin-mediated induction of angiogenesis has been found to derive partly from the upregulation of vascular endothelial growth factor receptor Flk-1/KDR by endothelial cells, through an integrin αvβ3-focal adhesion kinase-mediated signaling pathway. A recent report demonstrated that stromal periostin is a key limiting factor regulating lung metastasis of mouse breast tumors [30]. In human MCF-10A mammary breast cells, periostin-overexpression enhances tumor growth and metastasis to the lung [31].

Few studies on human breast cancer have revealed that periostin expression is considerably higher in cancer tissues than in normal tissues and is considered as tumor-enhancing factor [24–29]. Periostin expression is low or undetectable in normal breast epithelium [24, 25, 28, 29] and, in accordance with its known mesenchymal expression, it is expressed only in the stroma [28]. However, it is not clear whether periostin is produced and secreted by breast cancer cells rather than by cancer-associated stromal cells or by both. Several studies indicated that periostin overexpression appears to occur mainly in cancer-associated stroma [24, 26–28]. These findings contradict the report by Sho et al. [25]. Identifying epithelial breast cancer cells as the unique source of periostin expression. Puglisi et al. detected increased periostin expression in both cancer cells and cancer-associated stromal cells compared to normal tissue [26]. Although the reasons underlying these discrepancies are unclear, these findings could be explained by methodological differences, and different scoring systems and protein expression cut-off levels.

Although breast cancer is believed to develop from histologically identifiable intraductal lesions known as DCIS, there have been few studies examining periostin expression in breast tumor progression along the spectrum of normal-DCIS-IBC. Kharaishvili et al. evaluated periostin expression in 173 cases of invasive breast cancer, 36 cases of DCIS and 31 normal tissue samples (adjacent to a tumor) [28]. Periostin expression was either absent or low positive in IBC cancer cells, while highly positive in cancer-associated stroma in almost all cases. In normal tissue and DCIS, periostin expression was limited to the stromal compartment. Stromal and epithelial periostin expression was significantly higher in invasive breast cancer than in normal tissue and DCIS.

We also evaluated periostin expression in the epithelial compartment and the surrounding stromal tissue of samples in the following order: normal breast tissue, DCIS, and IBC. Normal breast tissue samples in this study were taken from women with no surgically proven pathological breast lesions and were not taken from normal tissue adjacent cancer. The sample size of DCIS in the present study was larger than that of the study by Kharaishvili et al. [28]. In the present study, stromal periostin SS in each normal breast tissue, DCIS, and IBC group was significantly higher than epithelial periostin SS. Moreover, stromal periostin SS significantly correlated with epithelial periostin SS in DCIS and IBC. These findings suggest that although periostin is secreted directly by tumor cells, stromal cells largely contribute to periostin secretion both in normal and in neoplastic conditions and an autocrine/paracrine periostin loop between stromal and epithelial tumor cells may exist.

The present study found changes in epithelial and stromal periostin expression during breast tumor progression, supporting the findings by Kharaishvili et al. [28] and suggest that periostin may play a different biological role in breast tumor progression, according to its compartmentalization. Epithelial periostin expression was not detected in normal breast tissues, although it was present to a variable extent in DCIS and IBC. Epithelial periostin expression was found to progressively increase from normal breast to IBC. The EMT participates in tumor progression by increasing cancer cell motility, migration, invasion, and adhesion and is characterized by upregulation of mesenchymal proteins, such as vimentin, fibronectin, and N-cadherin, and downregulation of epithelial markers, such as E-cadherin [12–14]. Periostin is a mesenchymal component, therefore, increased periostin expression in cancer cells may reflect the acquisition of EMT and is an early event in breast tumorigenesis. In the present study, although the difference in SS between normal and DCIS were not significant, stromal SS increased significantly from normal breast tissue with disease progression, in a stepwise fashion. Higher stromal SS was observed in IBC compared to normal tissue or DCIS. Furthermore, stromal periostin staining in IBC was more diffuse and intense and not simply juxtaposed to malignant epithelium, as seen in DCIS lesions. The alteration of stromal periostin expression suggests that the upregulation and redistribution of stromal periostin expression may be key events in the progression from DCIS to IBC.

A number of studies have examined the association between periostin expression and prognostic tumor features in patients with breast cancer [24, 26–28], although few studies have assessed the association between periostin expression and breast cancer prognosis. [29].

Puglisi et al. [26] evaluated periostin expression in 206 breast carcinomas and found that periostin expression was localized to the nucleus and/or cytoplasm of carcinoma cells, in addition to the stroma. Periostin expression in the cytoplasm or nucleus of carcinoma cells correlated with tumor size and expression of PR. Quantitative analysis of the periostin immunohistochemistry indicated that there was a trend of increased periostin expression from stage I, and II, to stage III and IV tumors [27]. Although serum periostin levels were elevated in patients with breast cancer presenting with bone metastases, compared to patients with breast cancer without evidence of bone metastasis [24], Kharaishvili et al. did not find an association between stromal periostin expression and bone metastasis [28].

To the best of our knowledge, only one study has directly compared periostin expression in breast cancer with clinical outcome, namely disease-specific survival [29]. Xu et al. performed an immunohistochemical evaluation of periostin expression in 1,086 invasive carcinomas. Periostin was located in the cytoplasm and membrane of the breast cancer cells. Periostin expression was classified semi-quantitatively according to the following criteria: 0 if <1% of neoplastic cells expressed periostin; 1+ if ≥1% and < 10% of neoplastic cells expressed periostin; and, 2+ if ≥10% of neoplastic cells discretely expressed periostin. Samples scored as 1+ or 2+ were considered positive. Positive periostin expression in cancer cells was observed in 334 (30.76%) of the 1,086 breast cases and correlated with tumor size, histological grade, lymph node metastasis, triple-negative breast cancer, and postoperative distant metastasis. Furthermore, positive epithelial periostin expression was associated with poorer disease-specific survival on univariate and multivariate analysis.

When comparing clinicopathological factors between the periostin high expression group and low expression group in cancer cells and cancer-associated stroma, there was no significant difference between the two groups, except for metastatic relapse. High epithelial periostin expression in IBC was more frequent in the distant metastatic relapse-positive group than in the distant metastatic relapse-negative group. The patients with high epithelial periostin expression had a significantly poorer prognosis for disease-free and overall survival than those with low epithelial expression. Furthermore, epithelial periostin expression was one of the independent prognostic factors for disease-freeand overall survival. Despite the high stromal predominance of periostin, stromal periostin expression did not correlate with survival of IBC patients. Our findings support those of Xu et al. [29] and suggest that epithelial expression of periostin may be associated with a more aggressive tumor phenotype in IBC and may be considered an essential biomarker of breast carcinoma for predicting the prognosis and identifying patients who may benefit the most from adjuvant treatment.

There are some limitations to our study: the retrospective nature of the study, the relatively small sample size, and the fact that it involved a single institution. In fact, there is a possibility that no standardized scoring method to categorize periostin expression and stromal cell heterogeneous population affects the result of this study. A prospective study involving a large number of cases is necessary in order to overcome these limitations and evaluate the predictive and prognostic value of periostin expression in breast carcinoma.

Upregulation of periostin in breast cancer tissues compared to normal tissues and correlation with postoperative distant metastasis and poor prognosis in patients with breast cancer suggests that periostin may be a potential target for therapeutic intervention in the future. Developing a further understanding of the cellular and molecular mechanisms by which periostin promotes tumor progression and identification of periostin functional modulators may allow the development of new strategies to treat breast cancer.

In conclusion, our findings indicate that breast tumor progression is accompanied by increased periostin expression. High periostin expression in cancer cells may be a novel parameter for the prediction of prognosis in patients with IBC. However, further studies are needed to clarify the role of periostin in breast cancer progression and metastasis.

## Supporting information

S1 File **Figure A. Periostin expression in normal breast tissue TMA cores.** Positive staining was restricted to the stromal connective tissue, whereas the epithelial component was devoid of periostin. Periostin was faintly expressed and confined to a narrow faint band adjacent to ducts (A, low magnification; B, high magnification). In some cases, normal ducts were surrounded by thick, well-defined bundles of stromal connective tissue (C, low magnification; D, high magnification). **Figure B. Periostin expression in ductal carcinoma in situ TMA cores.** There were discrete regions of periostin-positive stroma immediately adjacent to the malignant epithelium (A, low magnification; B, high magnification; C, low magnification; D, high magnification). Immunoreactivity of malignant epithelium was located in the cytoplasm and weaker than that of stromal compartment (C and D). **Figure C. Periostin expression in invasive breast carcinoma TMA cores.** Stromal periostin staining was diffuse and intense, while the epithelial component showed variable staining (A, low magnification; B, high magnification; C, low magnification; D, high magnification).
(PDF)
