# Supplementary material for: Epithelial periostin expression is correlated with poor survival in patients with invasive breast carcinoma
Source: PLoS One. 2017 Nov 21;12(11):e0187635. doi: 10.1371/journal.pone.0187635 (PMC5697858; doi:10.1371/journal.pone.0187635)

Figure A. Periostin expression in normal breast tissue TMA cores. Positive staining was restricted to the stromal connective tissue, whereas the epithelial component was devoid of periostin. Periostin was faintly expressed and confined to a narrow faint band adjacent to ducts (A, low magnification; B, high magnification). In some cases, normal ducts were surrounded by thick, well-defined bundles of stromal connective tissue (C, low magnification; D, high magnification).

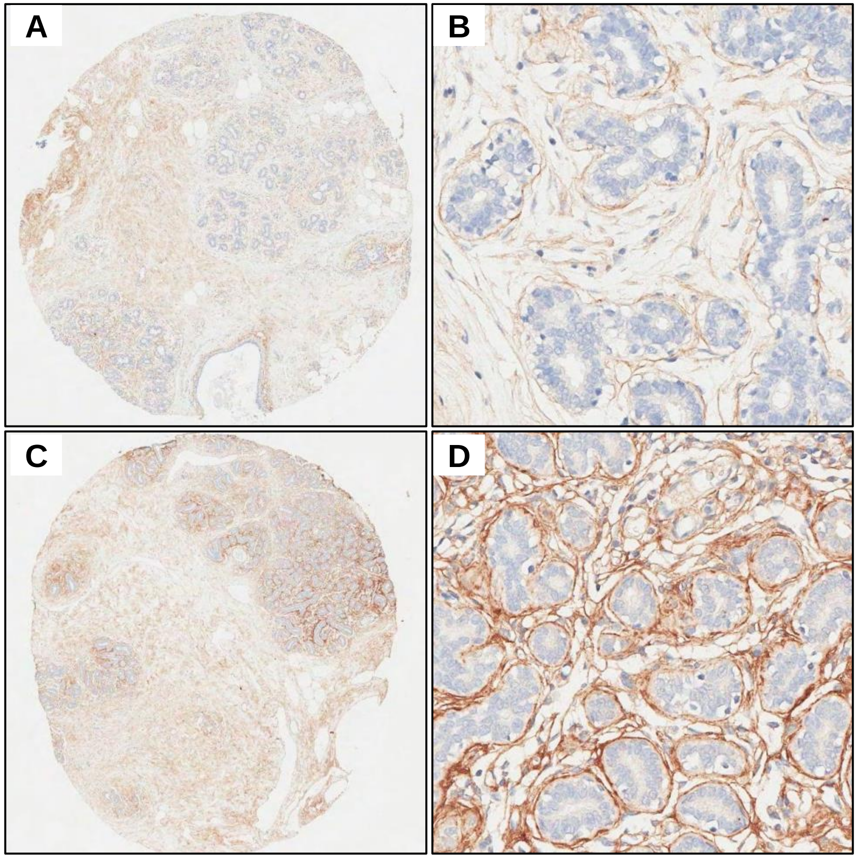

Figure B. Periostin expression in ductal carcinoma in situ TMA cores. There were discrete regions of periostin-positive stroma immediately adjacent to the malignant epithelium (A, low magnification; B, high magnification; C, low magnification; D, high magnification). Immunoreactivity of malignant epithelium was located in the cytoplasm and weaker than that of stromal compartment (C and D).

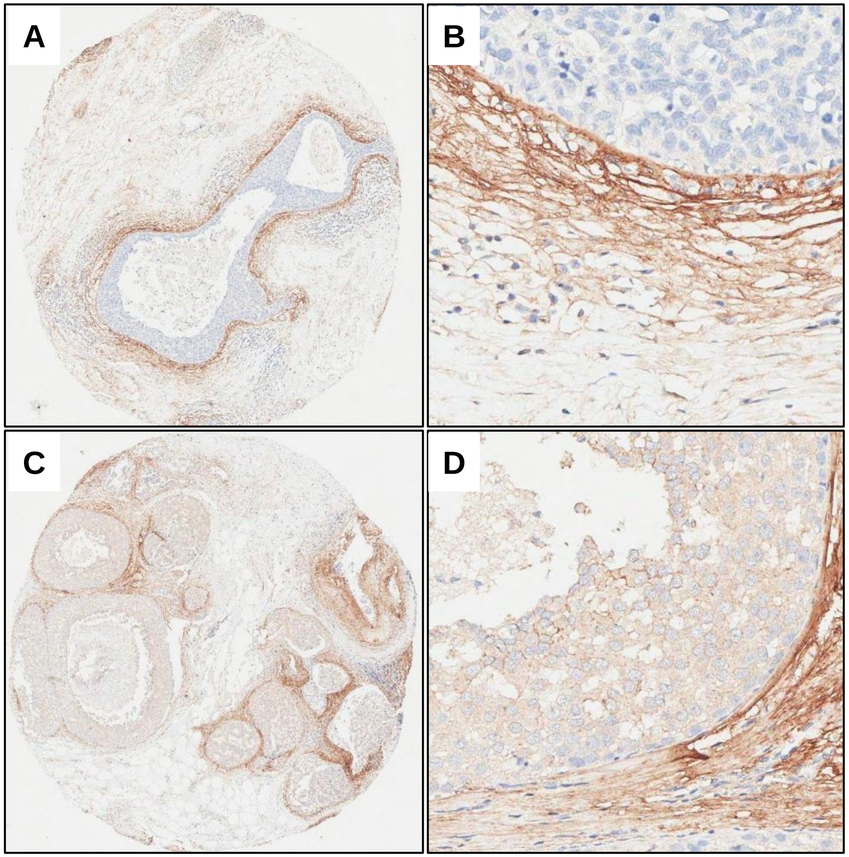

Figure C. Periostin expression in invasive breast carcinoma TMA cores. Stromal periostin staining was diffuse and intense, while the epithelial component showed variable staining (A, low magnification; B, high magnification; C, low magnification; D, high magnification).

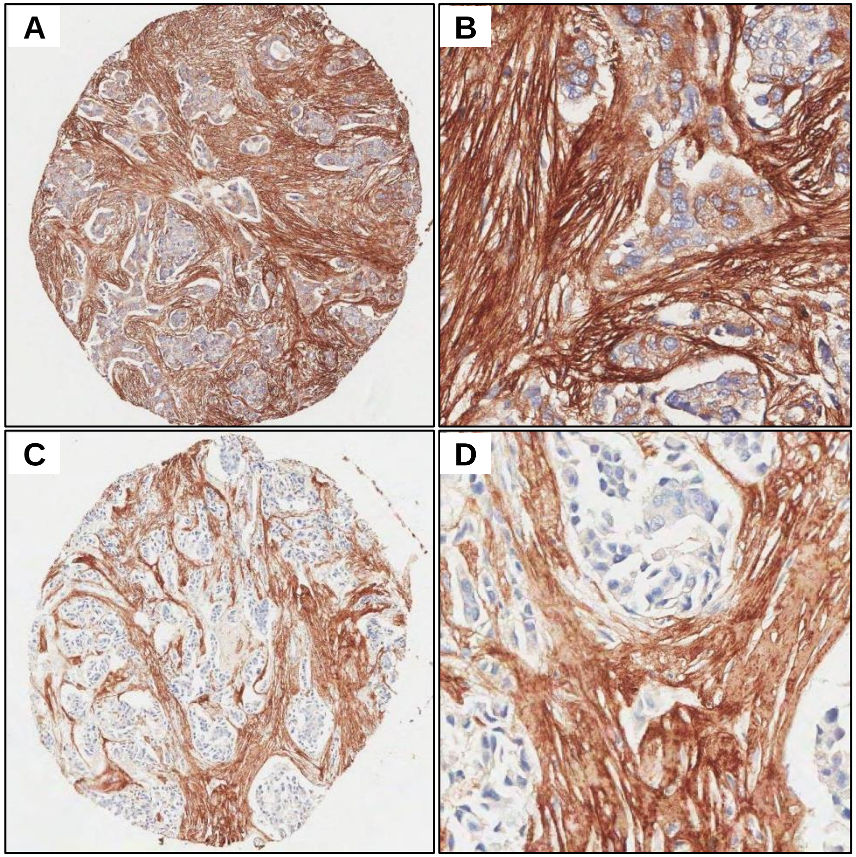

Supplement: S1 File — Figure A. Periostin expression in normal breast tissue TMA cores. Positive staining was restricted to the stromal connective tissue, whereas the epithelial component was devoid of periostin. Periostin was faintly expressed and confined to a narrow faint band adjacent to ducts (A, low magnification; B, high magnification). In some cases, normal ducts were surrounded by thick, well-defined bundles of stromal connective tissue (C, low magnification; D, high magnification). Figure B. Periostin expression in ductal carcinoma in situ TMA cores. There were discrete regions of periostin-positive stroma immediately adjacent to the malignant epithelium (A, low magnification; B, high magnification; C, low magnification; D, high magnification). Immunoreactivity of malignant epithelium was located in the cytoplasm and weaker than that of stromal compartment (C and D). Figure C. Periostin expression in invasive breast carcinoma TMA cores. Stromal periostin staining was diffuse and intense, while the epithelial component showed variable staining (A, low magnification; B, high magnification; C, low magnification; D, high magnification). (PDF) [file pone.0187635.s001.pdf]
